# 7,9-Bis(hy­droxy­meth­yl)-7*H*-purine-2,6,8(1*H*,3*H*,9*H*)trione

**DOI:** 10.1107/S1600536811018186

**Published:** 2011-05-20

**Authors:** M. Daudon, D. Bazin, K. Adil, A. Le Bail

**Affiliations:** a Laboratoire de Biochimie A, AP-HP, Hopital Necker, 149 rue de Sèvres, 75743 Paris Cedex 15, France; b Laboratoire de Physique des Solides, Bat. 510, Université Paris XI, 91045 Orsay, France; c Laboratoire des Oxydes et Fluorures, UMR 6010 CNRS, Université du Maine, Avenue Olivier Messiaen, 72085 Le Mans Cedex 9, France

## Abstract

The structure of the title uric acid derivative, C_7_H_8_N_4_O_5_, from human kidney stones, is characterized by the C and O atoms of one of the two hy­droxy­methyl groups being disordered nearly equally over three different sites. In the crystal, mol­ecules are connected by a three-dimensional hydrogen-bonding scheme though they look stacked in planes nearly parallel to (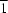
04).

## Related literature

For related structures, see: Ringertz (1966 ▶) for uric acid and Parkin & Hope (1998 ▶) for the dihydrate. For urolithia­sis, see: Tanagho & McAninch (2000 ▶); Jungers *et al.* (2005 ▶); Moe (2006 ▶); Knoll (2007 ▶). For recent characterization of new urinary stones, see: Le Bail *et al.* (2009 ▶); For purine biosynthesis, see: Ashihara *et al.* (2008 ▶). For hy­droxy­methyl­ation of uric acid, see: Lubczak *et al.* (2002 ▶).
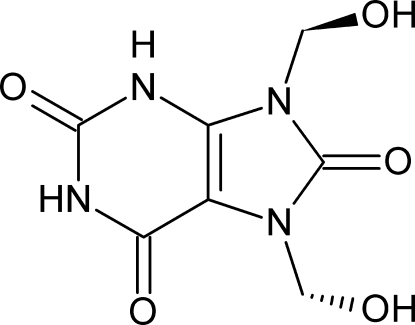


## Experimental

### 

#### Crystal data

C_7_H_8_N_4_O_5_
*M* *_r_* = 228.17
Monoclinic, 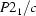

*a* = 5.3226 (6) Å
*b* = 11.5541 (13) Å
*c* = 14.5931 (18) Å
β = 97.340 (7)°
*V* = 890.09 (18) Å^3^
*Z* = 4
Mo *K*α radiation
μ = 0.15 mm^−1^
*T* = 150 K
0.22 × 0.12 × 0.06 mm

#### Data collection

Bruker Kappa APEXII CCD diffractometer
Absorption correction: multi-scan (*SADABS*; Bruker, 2008 ▶) *T*_min_ = 0.677, *T*_max_ = 0.746
33302 measured reflections
3075 independent reflections
2127 reflections with *I* > 2σ(*I*)
*R*_int_ = 0.064

#### Refinement

*R*[*F* ^2^ > 2σ(*F* ^2^)] = 0.056
*wR*(*F* ^2^) = 0.166
*S* = 1.03
3075 reflections
169 parameters
1 restraint
H-atom parameters constrained
Δρ_max_ = 0.78 e Å^−3^
Δρ_min_ = −0.37 e Å^−3^

### 

Data collection: *APEX2* (Bruker, 2008 ▶); cell refinement: *SAINT* (Bruker, 2008 ▶) and *McMaille* (Le Bail, 2004 ▶); data reduction: *SAINT*; program(s) used to solve structure: *SHELXS97* (Sheldrick, 2008 ▶) and *ESPOIR* (Le Bail, 2001 ▶); program(s) used to refine structure: *SHELXL97* (Sheldrick, 2008 ▶); molecular graphics: *DIAMOND* (Brandenburg, 2001 ▶) and *ORTEP-3* (Farrugia, 1997 ▶); software used to prepare material for publication: *publCIF* (Westrip, 2010 ▶).

## Supplementary Material

Crystal structure: contains datablocks I, global. DOI: 10.1107/S1600536811018186/zl2370sup1.cif

Structure factors: contains datablocks I. DOI: 10.1107/S1600536811018186/zl2370Isup2.hkl

Supplementary material file. DOI: 10.1107/S1600536811018186/zl2370Isup3.mol

Supplementary material file. DOI: 10.1107/S1600536811018186/zl2370Isup4.cml

Additional supplementary materials:  crystallographic information; 3D view; checkCIF report

## Figures and Tables

**Table 1:** Hydrogen-bond geometry (Å, °)

*D*—H⋯*A*	*D*—H	H⋯*A*	*D*⋯*A*	*D*—H⋯*A*
O9—H9⋯O2^i^	1.04	1.70	2.7270 (18)	169
N1—H1⋯O6^ii^	0.86	1.98	2.8388 (18)	179
N3—H3⋯O8^iii^	0.84	1.88	2.7104 (19)	167
O71—H71⋯O2^iv^	0.84	2.04	2.873 (4)	172
O72—H72⋯O9^i^	0.84	2.01	2.834 (5)	169
O73—H73⋯O8^v^	0.84	2.17	2.892 (6)	144

Symmetry codes: (i) 
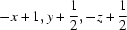
; (ii) 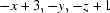; (iii) 
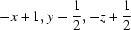
; (iv) 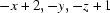; (v) 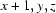.

**Fig. 1.**
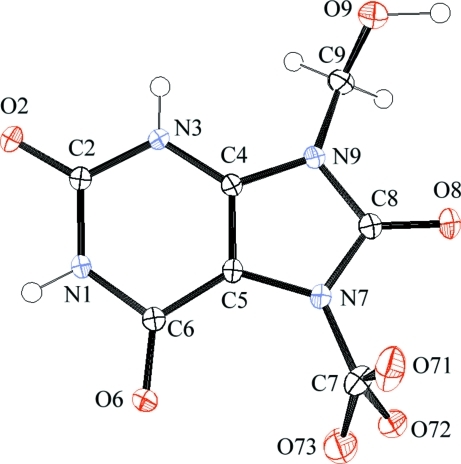
ORTEP view (Farrugia, 1997) of the title molecule; displacement ellipsoids are drawn at the 50% probability level. The nine hydrogen atoms bonded to C7, O71, O72 and O73, with ~1/3 occupancies are omitted for the sake of clarity.

**Fig. 2.**
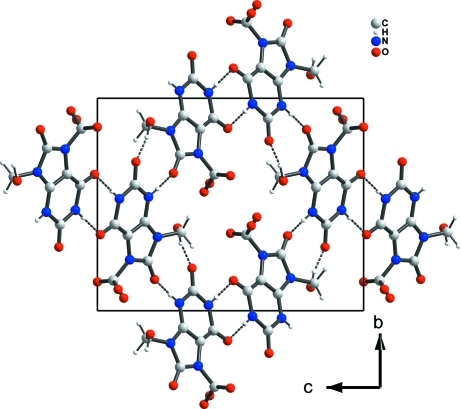
DIAMOND (Brandenburg, 2001) projection of the structure along the a axis. The N1—H1···O6, N3—H3···O8 and O9—H9···O2 hydrogen bonds ensuring the formation of layers are represented by dashed lines. H atoms with ~1/3 occupancy are not represented.

**Fig. 3.**
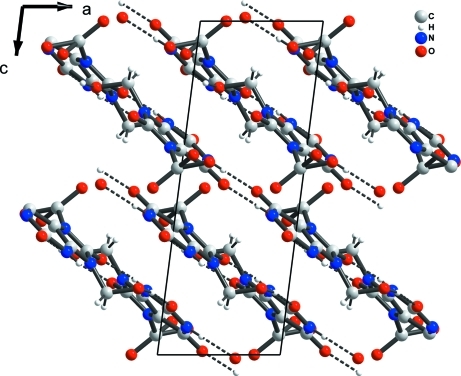
DIAMOND (Brandenburg, 2001) projection of the structure along the b axis showing the layers stacked parallel to (104). H atoms with ~1/3 occupancy are not represented. The disordered OH group (O71, O72, O73) is mainly involved in inter-layers hydrogen bonding.

## supplementary crystallographic information

### Comment

Urolithiasis, which is as old as mankind is now the third most common urinary disease (Jungers *et al.*, 2005; Moe, 2006; Knoll, 2007). This disease constitutes a major health problem and there is evidence to show that its incidence has increased continually in past decades (Tanagho & McAninch, 2000). Among the different chemical phases found in kidney stones, let's quote calcium oxalate, calcium phosphate, uric acid, ammonium hydrogen urate and magnesium ammonium phosphate which are the main components of stones, with differences in their distribution being found in different population groups.

Recently, at the surface of uric acid kidney stones, a green deposit has been observed for different patients. Since classical FTIR measurements were not able to characterize such deposit, X-ray diffraction experiments have been performed.

Powder diffraction revealed a mixture of uric acid (Ringertz, 1966) together with traces of its dihydrate (Parkin & Hope, 1998) and an unknown phase which could be indexed by using the McMaille software (Le Bail, 2004). An hypothesis for an uric acid derivative was suggested by the direct space software ESPOIR (Le Bail, 2001), however the structure could not be completed till a tiny single-crystal was selected in the powder. From the structure solution, a hydroxymethyl group was found attached to N9. High thermal parameters at room temperature obscured the nature of some disorder occurring around of the C7 atom: three peaks on the Fourier difference map, all looking lighter than a C atom, but heavier than a H one, two of them at 0.9 Å from each other were observed. At 150 K, the thermal motions were considerably smaller, allowing to propose an interpretation: a second hydroxymethyl group, CH_2_OH attached to N7, nearly equally disordered over three different O atom sites. The largest difference densities (0.78, 0.54) in the final structural model are close to C9 of the not disordered hydroxymethyl group (exactly between H9A and H9B) and O8. If O8 may be slightly splitted, given its large U_33_, the most intense residue close to C9 is unclear, possibly due to some disorder also for this hydroxymethyl group. Positional disorder was observed in uric acid dihydrate with superimposition of the six- and five-membered rings (Parkin and Hope, 1998). Such a disorder is unlikely to occur in the title compound. Different parts of the samples examined by powder diffraction may show variations in cell parameters as well as strong peak asymetries suggesting inhomogeneities, possibly corresponding to more or less disorder.

The *ORTEP* diagram of the title compound is shown in Fig. 1. Atoms numbering adopts the purine system. Molecules are connected by a three-dimensional hydrogen bonding scheme (Fig. 2 and Table 2), though they are stacked in planes nearly parallel to (104) (Fig. 3), corresponding by far to the most intense reflection.

In humans, uric acid is the main urinary metabolite of purines, therefore, its alteration is a mark of disorders associated with purine metabolism. A review on the biosynthesis of caffeine and related purine alkaloids was published recently (Ashihara *et al.*, 2008). But how the title compound is biosynthesized in humans is not yet fully understood. Hydroxymethylation of uric acid is known to occur with formaldehyde (Lubczak *et al.*, 2002).

### Experimental

Samples are coming from human kidney stones, always identified as a green part at the surface of uric acid calculi. Either they could originate from a natural cause or from the consequence of a chemical treatment of the patients (about ten cases). Since the patients took various medications for different unsimilar pathologies or had no treatment at all, the cause looks more probably natural.

### Refinement

H atoms of the N—H and 9-hydroxymethyl groups were positioned from the difference Fourier map. Owing to the disorder, the H atoms bonded to C7, O71, O72 and O73 were positioned geometrically (C—H = 0.99 and O—H = 0.84 Å). All H atoms were constrained to ride on their parent atoms with *U*_iso_(H) values set at 1.2 *x U*eq(C or N) and 1.5 *x U*eq(O). When refined independently, the occupancies of the three sites O71, O72 and O73 were very similar and their sum was very close to one. In the final model, the sum was constrained to one.

### Crystal data

**Table:**

C_7_H_8_N_4_O_5_	*F*(000) = 472.0
*M**_r_* = 228.17	*D*_x_ = 1.703 Mg m^−^^3^
Monoclinic, *P*2_1_/*c*	Mo *K*α radiation, λ = 0.71073 Å
Hall symbol: -P 2ybc	Cell parameters from 241 reflections
*a* = 5.3226 (6) Å	θ = 4–15°
*b* = 11.5541 (13) Å	µ = 0.15 mm^−^^1^
*c* = 14.5931 (18) Å	*T* = 150 K
β = 97.340 (7)°	Fragment, pale-green
*V* = 890.09 (18) Å^3^	0.22 × 0.12 × 0.06 mm
*Z* = 4	

### Data collection

**Table:**

Bruker Kappa APEXII CCD diffractometer	3075 independent reflections
Radiation source: fine-focus sealed tube	2127 reflections with *I* > 2σ(*I*)
graphite	*R*_int_ = 0.064
ω scans; 30 settings	θ_max_ = 32.3°, θ_min_ = 3.5°
Absorption correction: multi-scan (*SADABS*; Bruker, 2008)	*h* = −7→7
*T*_min_ = 0.677, *T*_max_ = 0.746	*k* = −17→17
33302 measured reflections	*l* = −21→21

### Refinement

**Table:**

Refinement on *F*^2^	Primary atom site location: structure-invariant direct methods
Least-squares matrix: full	Secondary atom site location: difference Fourier map
*R*[*F*^2^ > 2σ(*F*^2^)] = 0.056	Hydrogen site location: inferred from neighbouring sites
*wR*(*F*^2^) = 0.166	H-atom parameters constrained
*S* = 1.03	*w* = 1/[σ^2^(*F*_o_^2^) + (0.0816*P*)^2^ + 0.5575*P*] where *P* = (*F*_o_^2^ + 2*F*_c_^2^)/3
3075 reflections	(Δ/σ)_max_ < 0.001
169 parameters	Δρ_max_ = 0.78 e Å^−^^3^
1 restraint	Δρ_min_ = −0.37 e Å^−^^3^

### Special details

**Table:**

Geometry. All e.s.d.'s (except the e.s.d. in the dihedral angle between two l.s. planes) are estimated using the full covariance matrix. The cell e.s.d.'s are taken into account individually in the estimation of e.s.d.'s in distances, angles and torsion angles; correlations between e.s.d.'s in cell parameters are only used when they are defined by crystal symmetry. An approximate (isotropic) treatment of cell e.s.d.'s is used for estimating e.s.d.'s involving l.s. planes.
Refinement. Refinement of *F*^2^ against ALL reflections. The weighted *R*-factor *wR* and goodness of fit *S* are based on *F*^2^, conventional *R*-factors *R* are based on *F*, with *F* set to zero for negative *F*^2^. The threshold expression of *F*^2^ > σ(*F*^2^) is used only for calculating *R*-factors(gt) *etc*. and is not relevant to the choice of reflections for refinement. *R*-factors based on *F*^2^ are statistically about twice as large as those based on *F*, and *R*- factors based on ALL data will be even larger.

### Fractional atomic coordinates and isotropic or equivalent isotropic displacement parameters (Å^2^)

**Table:**

	*x*	*y*	*z*	*U*_iso_*/*U*_eq_	Occ. (<1)
O2	1.0659 (2)	−0.20036 (10)	0.35524 (10)	0.0250 (3)	
O6	1.3617 (2)	0.14185 (10)	0.48924 (9)	0.0241 (3)	
O8	0.5565 (3)	0.35248 (12)	0.28547 (13)	0.0430 (4)	
O9	0.2229 (2)	0.11073 (11)	0.19866 (10)	0.0278 (3)	
H9	0.1080	0.1830	0.1860	0.042*	
N1	1.2092 (3)	−0.02650 (11)	0.41726 (10)	0.0174 (3)	
H1	1.3389	−0.0620	0.4458	0.021*	
N3	0.8444 (3)	−0.03560 (11)	0.30815 (10)	0.0175 (3)	
H3	0.7360	−0.0740	0.2739	0.021*	
N7	0.8876 (3)	0.26000 (12)	0.37849 (11)	0.0247 (3)	
N9	0.6403 (3)	0.15297 (12)	0.27429 (11)	0.0224 (3)	
C2	1.0404 (3)	−0.09345 (13)	0.35937 (11)	0.0167 (3)	
C4	0.8172 (3)	0.08053 (13)	0.32201 (11)	0.0162 (3)	
C5	0.9722 (3)	0.14401 (13)	0.38595 (11)	0.0165 (3)	
C6	1.1933 (3)	0.09272 (13)	0.43511 (11)	0.0161 (3)	
C8	0.6827 (4)	0.26569 (15)	0.31068 (14)	0.0273 (4)	
C9	0.4761 (3)	0.12956 (15)	0.18565 (13)	0.0236 (4)	
H9A	0.4850	0.1962	0.1435	0.028*	
H9B	0.5407	0.0606	0.1559	0.028*	
C7A	0.9677 (4)	0.35741 (15)	0.44018 (14)	0.0268 (4)	0.339 (3)
H7A1	1.1376	0.3428	0.4744	0.032*	0.339 (3)
H7A2	0.9734	0.4301	0.4045	0.032*	0.339 (3)
O71	0.7889 (9)	0.3644 (4)	0.4997 (3)	0.0325 (11)	0.339 (3)
H71	0.8173	0.3134	0.5407	0.049*	0.339 (3)
C7B	0.9677 (4)	0.35741 (15)	0.44018 (14)	0.0268 (4)	0.340 (6)
H7B1	0.8166	0.3902	0.4638	0.032*	0.340 (6)
H7B2	1.0823	0.3278	0.4938	0.032*	0.340 (6)
O72	1.0842 (9)	0.4407 (3)	0.4008 (3)	0.0244 (13)	0.340 (6)
H72	0.9764	0.4847	0.3717	0.037*	0.340 (6)
C7C	0.9677 (4)	0.35741 (15)	0.44018 (14)	0.0268 (4)	0.321 (6)
H7C1	0.9760	0.3322	0.5053	0.032*	0.321 (6)
H7C2	0.8424	0.4209	0.4298	0.032*	0.321 (6)
O73	1.2214 (10)	0.3999 (4)	0.4221 (4)	0.0344 (15)	0.321 (6)
H73	1.2569	0.3717	0.3723	0.052*	0.321 (6)

### Atomic displacement parameters (Å^2^)

**Table:**

	*U*^11^	*U*^22^	*U*^33^	*U*^12^	*U*^13^	*U*^23^
O2	0.0232 (6)	0.0108 (5)	0.0381 (7)	0.0017 (4)	−0.0069 (5)	−0.0015 (5)
O6	0.0215 (6)	0.0168 (5)	0.0301 (7)	0.0027 (4)	−0.0114 (5)	−0.0047 (5)
O8	0.0344 (8)	0.0159 (6)	0.0700 (11)	0.0077 (5)	−0.0270 (8)	−0.0004 (6)
O9	0.0188 (6)	0.0212 (6)	0.0422 (8)	−0.0021 (5)	−0.0010 (5)	0.0016 (5)
N1	0.0157 (6)	0.0121 (6)	0.0224 (7)	0.0027 (5)	−0.0051 (5)	0.0001 (5)
N3	0.0161 (6)	0.0106 (6)	0.0237 (7)	−0.0005 (5)	−0.0057 (5)	−0.0006 (5)
N7	0.0221 (7)	0.0125 (6)	0.0355 (8)	0.0039 (5)	−0.0117 (6)	−0.0033 (5)
N9	0.0200 (7)	0.0122 (6)	0.0313 (8)	0.0000 (5)	−0.0113 (6)	0.0023 (5)
C2	0.0152 (7)	0.0126 (6)	0.0211 (7)	0.0003 (5)	−0.0019 (6)	0.0008 (5)
C4	0.0135 (7)	0.0127 (6)	0.0209 (7)	−0.0001 (5)	−0.0030 (5)	0.0020 (5)
C5	0.0158 (7)	0.0113 (6)	0.0211 (7)	0.0019 (5)	−0.0027 (6)	0.0001 (5)
C6	0.0159 (7)	0.0134 (6)	0.0179 (7)	0.0010 (5)	−0.0016 (5)	−0.0001 (5)
C8	0.0231 (9)	0.0147 (7)	0.0397 (10)	0.0022 (6)	−0.0129 (8)	−0.0005 (7)
C9	0.0204 (8)	0.0193 (8)	0.0282 (9)	−0.0016 (6)	−0.0078 (7)	0.0027 (6)
C7A	0.0307 (10)	0.0149 (7)	0.0329 (9)	0.0015 (6)	−0.0031 (7)	−0.0041 (6)
O71	0.049 (3)	0.024 (2)	0.025 (2)	0.0127 (18)	0.0052 (18)	0.0001 (15)
C7B	0.0307 (10)	0.0149 (7)	0.0329 (9)	0.0015 (6)	−0.0031 (7)	−0.0041 (6)
O72	0.022 (2)	0.0179 (19)	0.032 (2)	−0.0022 (16)	0.0001 (16)	0.0008 (15)
C7C	0.0307 (10)	0.0149 (7)	0.0329 (9)	0.0015 (6)	−0.0031 (7)	−0.0041 (6)
O73	0.028 (3)	0.027 (2)	0.048 (3)	−0.003 (2)	0.002 (2)	0.005 (2)

### Geometric parameters (Å, °)

**Table:**

O2—C2	1.2450 (19)	N9—C4	1.380 (2)
O6—C6	1.2524 (19)	N9—C8	1.414 (2)
O8—C8	1.236 (2)	N9—C9	1.491 (2)
O9—C9	1.401 (2)	C4—C5	1.376 (2)
O9—H9	1.0371	C5—C6	1.426 (2)
N1—C2	1.387 (2)	C9—H9A	0.9900
N1—C6	1.406 (2)	C9—H9B	0.9900
N1—H1	0.8627	C7A—O71	1.371 (5)
N3—C4	1.3673 (19)	C7A—H7A1	0.9900
N3—C2	1.377 (2)	C7A—H7A2	0.9900
N3—H3	0.8402	O71—H71	0.8400
N7—C8	1.377 (2)	O72—H72	0.8400
N7—C5	1.414 (2)	O73—H73	0.8400
N7—C7A	1.470 (2)		
			
C9—O9—H9	114.0	C4—C5—C6	120.28 (14)
C2—N1—C6	127.52 (13)	N7—C5—C6	131.83 (14)
C2—N1—H1	116.4	O6—C6—N1	120.36 (14)
C6—N1—H1	116.1	O6—C6—C5	127.36 (14)
C4—N3—C2	118.90 (13)	N1—C6—C5	112.28 (13)
C4—N3—H3	121.9	O8—C8—N7	127.05 (17)
C2—N3—H3	118.8	O8—C8—N9	125.52 (16)
C8—N7—C5	108.41 (13)	N7—C8—N9	107.43 (14)
C8—N7—C7A	123.00 (14)	O9—C9—N9	112.21 (16)
C5—N7—C7A	127.88 (14)	O9—C9—H9A	109.2
C4—N9—C8	107.66 (13)	N9—C9—H9A	109.2
C4—N9—C9	127.92 (14)	O9—C9—H9B	109.2
C8—N9—C9	122.74 (14)	N9—C9—H9B	109.2
O2—C2—N3	122.35 (14)	H9A—C9—H9B	107.9
O2—C2—N1	121.10 (14)	O71—C7A—N7	105.2 (2)
N3—C2—N1	116.54 (13)	O71—C7A—H7A1	110.7
N3—C4—C5	123.92 (14)	N7—C7A—H7A1	110.7
N3—C4—N9	126.78 (14)	O71—C7A—H7A2	110.7
C5—C4—N9	109.29 (13)	N7—C7A—H7A2	110.7
C4—C5—N7	107.20 (13)	H7A1—C7A—H7A2	108.8

### Hydrogen-bond geometry (Å, °)

**Table:**

*D*—H···*A*	*D*—H	H···*A*	*D*···*A*	*D*—H···*A*
O9—H9···O2^i^	1.04	1.70	2.7270 (18)	169
N1—H1···O6^ii^	0.86	1.98	2.8388 (18)	179
N3—H3···O8^iii^	0.84	1.88	2.7104 (19)	167
O71—H71···O2^iv^	0.84	2.04	2.873 (4)	172
O72—H72···O9^i^	0.84	2.01	2.834 (5)	169
O73—H73···O8^v^	0.84	2.17	2.892 (6)	144

Symmetry codes: (i) −*x*+1, *y*+1/2, −*z*+1/2; (ii) −*x*+3, −*y*, −*z*+1; (iii) −*x*+1, *y*−1/2, −*z*+1/2; (iv) −*x*+2, −*y*, −*z*+1; (v) *x*+1, *y*, *z*.
